# Time estimation in a case of Tourette's syndrome: Effect of antipsychotic medications

**DOI:** 10.1002/npr2.12101

**Published:** 2020-03-15

**Authors:** Takuma Inagawa, Natsuki Ueda, Kazuyuki Nakagome, Tomiki Sumiyoshi

**Affiliations:** ^1^ Department of Psychiatry National Center Hospital National Center of Neurology and Psychiatry Tokyo Japan; ^2^ Department of Clinical Epidemiology Translational Medical Center National Center of Neurology and Psychiatry Tokyo Japan; ^3^ NCNP Brain Physiology and Pathology Tokyo Medical and Dental University Tokyo Japan; ^4^ Department of Preventive Intervention for Psychiatric Disorders National Center of Neurology and Psychiatry National Institute of Mental Health Tokyo Japan

**Keywords:** dopamine, time perception, Tourette's syndrome

## Abstract

**Aims:**

Dopamine (DA) hyperactivity causes overestimation of time, whereas DA hypoactivity produces its underestimation. DA activity also provides neurochemical substrates pertinent to several psychiatric conditions, such as schizophrenia and Tourette's syndrome. The overestimation of time sometimes exists in patients with Tourette's syndrome, but no reports have addressed time perception in relation to antipsychotic medications which typically act as DA receptor antagonists. We herein report a case of Tourette's syndrome, in which time estimation was differentially affected by risperidone (a DA antagonist) and aripiprazole (a DA partial agonist).

**Case:**

A 27‐year‐old man who suffered from verbal and motor tics was treated with risperidone. His tic symptoms disappeared; however, he began to experience a strange feeling that “time is going too fast.” For example, “people walk more quickly compared to a normal pace.” These complaints were thought to represent underestimation of time. Then, risperidone was switched to oral aripiprazole to optimize DA transmissions, which resulted in the amelioration of these subjective feelings.

**Conclusion:**

Our observations indicate that the underestimation of time may occur in patients with Tourette's syndrome who receive antipsychotic drugs with high DA D_2_ receptor blocking potency. This may support the concept that the estimation of time is influenced by DA activity.

## Abstract 2

Estimation of time in patients with Tourette's syndrome is affected by actions of antipsychotic drugs on dopamine type 2 receptors.

## Graphical abstract

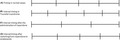

## 1 INTRODUCTION

The underestimation of time is represented by the feeling of “time going fast,” whereas its overestimation is described as “time going slow.” Dopamine (DA) hyperactivity causes the overestimation of time, whereas DA hypoactivity produces the underestimation of time.^1^, ^2^ The overestimation of time exists in some patients with Tourette's syndrome^3^ or schizophrenia.^4^ Tourette's syndrome is neurochemically characterized by altered mesocortical and mesostriatal DA transmissions.^3^, ^5^ At the anatomical level, there is an overabundance of DA nerve terminals in the striatum, which constitutes the DA hyperinnervation hypothesis^6^ of the disease. In this theory, the overabundance of DA nerve terminals is associated with an increase in DA synthesis and release via inputs from the midbrain to the striatum.^3^ As no reports have addressed time perception in relation to antipsychotic medications in patients with Tourette's syndrome, we report a case of this disorder in which time estimation was improved by a switch from risperidone (DA antagonist) to aripiprazole (DA partial agonist).

## 2 CASE REPORT

A 27‐year‐old man was suffering from uncomfortable feelings. He had no family history of Tourette's syndrome or physical comorbidities. At age six, he started calling out “Je [dʒə]” and “I [i],” spitting, and touching his body. Around the age of 10, his doctor told him that his symptoms would improve without any treatment by the age of 20.

After having graduated from university, he began working in sales to the age of 25, when he retired and started attending a school for comedy. At this time, his symptoms remained, so he visited another children's clinic, where he was diagnosed with Tourette's syndrome. He was prescribed 1 mg of oral risperidone, but his verbal and motor tics continued.

When he was 27, he decided to visit our hospital in April 2017. His doctor prescribed 1.5 mg oral risperidone, to be taken just before bed. Within 1 week, his family members reported that he stopped calling out “Je” and “I,” spitting, and touching his body. However, he still wanted to call out, in some occasions, especially when he felt stressed. After increasing the dose of risperidone to 2 mg, he was moved to another hospital because his doctor assessed that his tic symptoms were successfully treated. However, the symptoms reappeared one month later. In July 2017, his doctor increased the dose of risperidone to 2.5 mg. Thereafter, he experienced no tics.

In February 2018, he began to experience a strange feeling for approximately 15 minutes that people seemed to walk quickly, but then return to a normal pace. His family told him that people had been walking at a normal pace. He experienced this feeling at least twice a week for 6 months. This usually appeared when he looked at the window after he went to bed at night. He was still taking 2.5 mg of oral risperidone. In July 2018, he visited our hospital again to treat these symptoms. He also suffered from ejaculation difficulty. At that time, he was neither depressed nor manic.

In September 2018, the dose of risperidone was decreased to 1.5 mg and coadministration of 6 mg oral aripiprazole was initiated. From October 2018 onwards, he has no longer experienced the abnormal feeling of “time going fast.” His ejaculation disorder has also improved. On the basis of these observations, we gradually reduced the dose of risperidone, whereas the dose of aripiprazole was increased.

Since December 2018, he has been taking 15 mg of oral aripiprazole alone and has never experienced the feeling of “time is going too fast” again or suffered from tics. Currently, he is attending a school for comedy.

## 3 DISCUSSION

**FIGURE 1:**
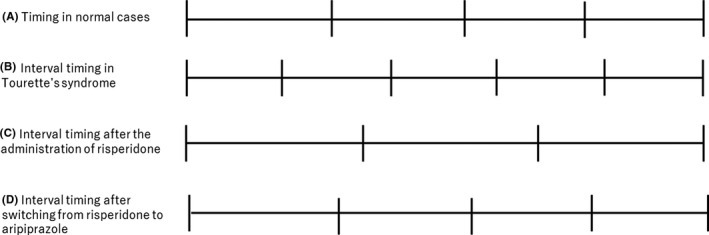
Interval timing in Tourette's syndrome in relation to antipsychotic treatments. A, Timing in normal cases. B, Interval timing in Tourette's syndrome. Patients with the disorder tend to overestimate time and feel that “time goes slowly.” C, Interval timing after the administration of risperidone. Antipsychotic drugs with a high dopamine‐D_2_ receptor blocking potency, such as risperidone, may cause overestimation of time, so the patients feel “time goes fast.” D, Interval timing after switching from risperidone to aripiprazole. Aripiprazole, a D_2_ receptor partial agonist, is less potent in blocking D_2_ receptor compared to risperidone, so that patients feel “time goes more slowly”

To our knowledge, this is the first report on a case of Tourette's syndrome in which the underestimation of time experienced during the administration of risperidone was relieved by a switch to aripiprazole. The complaint of “time is going fast,” subjectively perceived, refers to an underestimation of time as perceived by a patient, possibly caused by the blockade of DA D_2_ receptors in the striatum by risperidone, an antipsychotic drug with high potency for these receptors^6^ (Figure 1C). Patients with Tourette's syndrome may tend to underestimate time more clearly when they take antipsychotic drugs (Figure 1B). Because they actually overestimate time before they start taking antipsychotic medications (Figure 1A), they may feel more sensitive to the changes of time perception by antipsychotics.^3^ Time underestimation, experienced in this way, may have disappeared because of the switch from risperidone (a D_2_ receptor antagonist) to aripiprazole (a D_2_ receptor partial agonist). Aripiprazole, with its partial agonistic effect, may partially activate striatal DA transmissions (Figure 1D), contributing to the normalization of time perception.

A limitation of our study is that our observations were not based on objective assessments. Currently, there are some measurement scales to assess abnormal time perception. For example, the “Anticipation of Movement” task is designed to evaluate the ability of subjects to visually predict movement timing.^7^ However, no objective scales have been established for abnormal time perception in patients with Tourette's syndrome. To develop appropriate tasks may help objectively evaluate the effects of antipsychotic medications on abnormal time perception in patients with Tourette's syndrome.

In sum, our observations suggest that the underestimation of time sometimes occurs in patients with Tourette's syndrome who receive antipsychotic drugs with high D_2_ receptor blocking potency. This may support the concept that the estimation of time is influenced by DA activity.

## CONFLICT OF INTEREST

TI and NU declare no conflict of interest. TS reports personal fees from Otsuka Pharmaceuticals outside the submitted work. KN reports grants from research funding, personal fees from speaker's honoraria, and personal fees from attending an advisory board meeting from Otsuka Pharmaceuticals outside the submitted work.

## AUTHOR CONTRIBUTIONS

TI treated the patient and drafted the manuscript. NU, KN, and TS critically reviewed the draft and revised it. All authors made substantial contributions, drafted the manuscript, and approved the final manuscript.

## INFORMED CONSENT

The patient gave informed consent, and their anonymity has been preserved.

## Data Availability

The data that support the findings of this study are included in this article.
